# The Columbus, Georgia, Academy of Medicine

**Published:** 1899-03

**Authors:** T. E. Mitchell


### SOCIETY REPORTS.

---

### THE COLUMBUS, GEORGIA, ACADEMY OF MEDICINE.

At the regular meeting, January 27th, 1899, officers for the ensuing year were installed as follows: Dr. C. L. Williams, president; Dr. T. E. Mitchell, vice-president, and Dr. C. D. Wall, secretary and treasurer. Dr. W. T. Gautier was installed as a member of the executive committee, to serve a full term of three years.

At the regular semi-monthly meeting, on the evening of February 17th, Dr. W. J. Love, of Lafayette, Ala., read a paper which was the report of a case of "infection of gunshot wound of leg with the bacillus ærogenes capsulatus; amputation; recovery." The paper was supplemented by a report of the bacteriological findings in an examination of the amputated limb by Prof. C. A. Cary, of Auburn, Ala.

Beginning with the classical and comprehensive researches of Welch and Nutall, which appeared in the *Johns Hopkins Hospital Bulletin* for July and August, 1892, the essayist reviewed the literature of this interesting bacillus more or less completely to the present time. He called attention to the researches of Welch and Nutall, which recite that these investigators did an autopsy in the case of a man eight hours after death, which followed suddenly a severe hemorrhage, through openings in the anterior thoracic-wall, caused by a large sacculated aneurism of the ascending aorta. From the blood in the large vessels, heart, liver, spleen and kidneys, pure cultures of the bacillus were made. These tissues contained much gas, and the development of the bacillus in the culture media was manifested by the presence of gas in large quantities.

Morphologically, the bacillus ærogenes capsulatus is large, usually straight, with slightly rounded ends; it has a distinct capsule, is non-motile and does not form spores; it is from three to six micro-millimeters in length and about one-third as broad as long. It is strictly anerobic and stains with the aniline dyes, including Gram's method. It grows best at a temperature of thirty-five to thirty-seven degrees C. Subcutaneously, it is pathogenic for mice, guinea-pigs, rabbits and pigeons.

The bacillus develops rapidly with an abundant formation of gas throughout the body, in animals killed shortly after an intravenous injection.

The gas is odorless, and burns with a blue flame.

The essayist reviewed the clinical features of an obstetrical case reported by Graham, Stewart and Baldwin (*Columbus Medical Journal*, August, 1893), in which the woman, aged 35, shortly after a criminal abortion, was suddenly seized with a violent chill, which lasted four hours, followed by severe pelvic pains, with discharge of red blood from the uterus. Five hours after the chill there was extreme pain and restlessness. In another six hours she was emphysematous from head to foot, and died soon afterwards.

In the September, 1897, number of *Johns Hopkins Hospital Bulletin* Dr. Dobbins put on record the first case in which this bacillus was found ante-mortem. It was a case of contracted pelvis—large child with impacted head and protracted labor. The gas-producing bacilli were found in large numbers in a frothy, bloody fluid which escaped from the vagina; they were also found in the fetus and placenta. Shortly after death, the entire body became rapidly emphysematous.

The essayist cited these cases as being of interest to obstetricians as well as to surgeons.

He quoted from the work of Menge and Kronig (1897) on the "Bacteriology of the Female Genital Organs," to show that these authors attach great importance to infection with this bacillus.

The essayist referred to an article by Dr. Erdman (*Medical News*, October, 1897), in which he made a summary of all surgical cases previously published by Welch and Flexner and by Dunham, together with his own cases. From this summary the essayist finds that there had been published to that time sixteen surgical cases, twelve of which died and four recovered. Of the sixteen cases, fourteen were of mixed infection, six with the colon bacillus, and eight with the ordinary pus-producing germs. Only two of the sixteen cases were of pure infection. The four cases that recovered were of mixed infection, and amputation was done in each instance. From a study of all the cases reported, the essayist deduced the following observations: Infection with the bacillus ærogenes capsulatus produces a rapid toxemia, with a rapid breaking down of the infected tissues, and accumulation of gas in the same, rapidly spreading gangrene, and death, unless very prompt and thorough means are used for the removal of the entire infected area. Amputation, when practicable, is always indicated.

The following are some of the salient points of the case reported by the essayist: Saw a colored man forty-eight hours after he had been shot in the right leg below the knee while in a state of intoxication; status præsens; entire leg enormously swollen; wound of entrance in fairly good condition; wound of exit ugly and discharging a frothy fluid on slight pressure; the skin all over the limb gave a crackling sensation under pressure peculiar to emphysema; a peculiar fetid odor emanated from the limb. Patient complained of intense pain, and was extremely prostrated. Temperature 102; respiration 26; pulse 135, weak and irregular. For twenty-four hours after receiving the wound, patient was fairly comfortable. At this time a severe chill of several hours' duration was followed by pain, nausea, etc. After the usual preparations a deep incision was made in the limb, but no pus was found; but instead, there escaped a frothy, brownish fluid that spluttered with a bubbling noise which was distinctly audible several feet away, accompanied by a horrible odor which filled the room. All necrotic tissue was removed, the parts well irrigated, and an antiseptic toilet made, after which the patient was put to bed in a fairly good condition. Smear preparations from the scrapings and from the blood in the tibial artery showed a large, straight bacillus with rounded ends in great numbers. In view of the clinical history, the essayist supposed this to be the bacillus ærogenes capsulatus of Welch and Nutall, which supposition was confirmed by subsequent investigations. Next morning patient very restless; respiration 28; temperature 102; pulse 130. Much pain about the knee, but none below. Below the dressing, leg much swollen, emphysematous, and entirely without sensation. Amputation was advised and done the same day at the apex of Scarpa's triangle. With a few unimportant interruptions, the patient made a good recovery.

The report of the bacteriological examination of the amputated leg by Prof. C. A. Cary was very exhaustive and comprehensive. It showed a micrococcus and a bacillus which was substantially identical with the bacillus ærogenes capsulatus of Welch and Nutall.

The following from the report is very interesting: In the jugular vein of a rabbit was injected 2cc. of water from a potato culture, and the animal left in room temperature for twenty-two hours, during which time its abdomen became greatly distended, with considerable edema beneath the skin. The gas from the abdomen burned with a blue flame. The liver was greatly distended, with large bubbles of gas in its structure and under its capsule. Small pieces of the liver floated in 50 per cent. alcohol, and sections stained with Gram's method showed great numbers of the bacilli lining the gas spaces in the organ.

The essayist pointed out that this was the third case of pure infection by this specific germ that had been reported, and the only case of pure infection that had recovered, the other four cases of reported recovery having been of mixed infection.

T. E. MITCHELL, M.D.

